# Electrocardiogram monitoring as a predictor of neurological and survival outcomes in patients with out-of-hospital cardiac arrest: a single-center retrospective observational study

**DOI:** 10.3389/fneur.2023.1210491

**Published:** 2023-07-04

**Authors:** Masaki Takahashi, Kentaro Ogura, Tadahiro Goto, Mineji Hayakawa

**Affiliations:** ^1^Division of Acute and Critical Care Medicine, Department of Anaesthesiology and Critical Care Medicine, Hokkaido University Faculty of Medicine, Sapporo, Japan; ^2^Faculty of Medicine, The University of Tokyo, Tokyo, Japan

**Keywords:** out-of-hospital cardiac arrest, electrocardiogram, machine learning, outcome prediction, neurological outcomes, resuscitation

## Abstract

**Introduction:**

This study hypothesized that monitoring electrocardiogram (ECG) waveforms in patients with out-of-hospital cardiac arrest (OHCA) could have predictive value for survival or neurological outcomes. We aimed to establish a new prognostication model based on the single variable of monitoring ECG waveforms in patients with OHCA using machine learning (ML) techniques.

**Methods:**

This observational retrospective study included successfully resuscitated patients with OHCA aged ≥ 18 years admitted to an intensive care unit in Japan between April 2010 and April 2020. Waveforms from ECG monitoring for 1 h after admission were obtained from medical records and examined. Based on the open-access PTB-XL dataset, a large publicly available 12-lead ECG waveform dataset, we built an ML-supported premodel that transformed the II-lead waveforms of the monitoring ECG into diagnostic labels. The ECG diagnostic labels of the patients in this study were analyzed for prognosis using another model supported by ML. The endpoints were favorable neurological outcomes (cerebral performance category 1 or 2) and survival to hospital discharge.

**Results:**

In total, 590 patients with OHCA were included in this study and randomly divided into 3 groups (training set, *n* = 283; validation set, *n* = 70; and test set, *n* = 237). In the test set, our ML model predicted neurological and survival outcomes, with the highest areas under the receiver operating characteristic curves of 0.688 (95% CI: 0.682–0.694) and 0.684 (95% CI: 0.680–0.689), respectively.

**Conclusion:**

Our ML predictive model showed that monitoring ECG waveforms soon after resuscitation could predict neurological and survival outcomes in patients with OHCA.

## 1. Introduction

Despite the advances in cardiopulmonary resuscitation (CPR) and postcardiac arrest management, out-of-hospital cardiac arrest (OHCA) is associated with poor prognosis, particularly in terms of poor neurological outcomes. Worldwide, the incidence rate of OHCA ranges from 30.0 to 97.1 per 100,000 person-years, and the survival rate ranges from 3.1 to 20.4% (1). Due to severe hypoxic-ischemic brain injury, two-thirds of the patients with OHCA die before hospital discharge after resuscitation (2), and 2.8–18.2% have favorable neurological outcomes (1). An adequate neurological prognosis is essential for clinicians and patients’ families to decide whether or not to continue life-sustaining therapies.

Many prognostic scores have been developed using relevant variables to predict the prognosis of patients with OHCA (3–6). These variables include various types of information: basic characteristics of patients before cardiac arrest (CA; age, sex, and comorbidities), CA situations (witnessed and bystander CPR status, location of CA, cause of CA, and initial rhythm), clinical examinations (pupillary diameter, pupillary reflex to light, and the Glasgow Motor Score), and biomarkers after resuscitation. However, electrocardiogram (ECG) findings have not been used as a predictor, and few studies have investigated the relationship between ECG and outcomes in patients with OHCA (7–10).

Previous studies have reported that ECG can predict mortality in acute coronary syndromes (11), acute pulmonary embolism (12), sepsis (13), and trauma (14). In the patients with OHCA, some studies have analyzed ECG waveforms as predictors. These studies focused on a single component in ECG waveforms, such as heart rate (7), heart rate variability (HRV) (8), ST segment (9), and QT interval (10). Specifically, studies examining neurological outcomes in patients with OHCA have used heart rate (7) or HRV (8) as factors because the heart rate reflects the function of autonomic nervous system. In this study, we attempted to analyze various components of the waveform of continuous ECG monitoring using machine learning (ML) and proposed a novel predictor for the patients with OHCA.

## 2. Materials and methods

### 2.1. Study design, setting, and ethical approval

This was a retrospective cohort study of adult patients (age ≥ 18 years) admitted to the intensive care unit (ICU) of Hokkaido University Hospital for OHCA between April 2010 and April 2020. Hokkaido University Hospital is a tertiary care hospital that accepts approximately 1,000 critically-ill patients annually, including OHCA. We followed the transparent reporting of a multivariate prediction model for individual prognosis or diagnosis statement (15) for prognostic studies. This study was approved by the Ethics Committee of Hokkaido University Hospital (approval number: 019–0438). The committee agreed to waive the need for informed consent because this was a retrospective observational study that involved de-identification of the personal data of the patients.

### 2.2. Study participants and monitored data

We included adult patients with OHCA aged ≥ 18 years who were admitted to the ICU based on the in-hospital OHCA registry. We extracted the monitored time-series data, including the ECG data acquired by the ICU monitor device (IntelliVue MP70, Phillips Japan, Tokyo, Japan). Of several leads, only lead II waveforms of the ECG were analyzed in this study. Because the monitoring ECG waveforms are likely unstable at admission to the ICU due to procedures or position changes, we used 1-h monitoring ECG waveforms from 1 h after admission to the ICU for analysis. The exclusion criteria were the patients: (1) whose all acquired values were zero and (2) among whom no ECG waveform was detected between 1 and 2 h from the beginning of monitoring at ICU admission.

### 2.3. Outcomes

The primary outcome was in-hospital all-cause mortality and the secondary outcome was a favorable neurological outcome at discharge based on the Cerebral Performance Categories (CPC) score (16). A favorable neurological outcome was defined as a CPC score of 1 or 2.

### 2.4. Statistical analyses

The analytical processes were as follows: (1) preprocessing of ECG wave data monitoring, (2) premodelling with publicly available ECG data, and (3) modelling development and internal validation.

### 2.5. Monitoring-ECG data preprocessing

The distribution of values (μV) while monitoring the ECG data is shown in Supplementary Table 1. Most values were in the range of 7,000–9,000 μV, while some were significantly below or above the range. These were considered abnormal values due to body movement, medical procedures, and preparation for room transfer; therefore, we excluded them from the analysis. Data with constant values longer than 1 min were also excluded. The periodicity of the ECG data was analyzed by obtaining the time at the top of the R-wave using BioSPPy (17). The ECG waveform was digitized at a frequency of 500 data points per second.

### 2.6. Premodelling with PTB-XL ECG data

To develop the prediction model efficiently, we used open-access PTB-XL data (18) for premodelling. PTB-XL is a large dataset of 10-s 12-lead ECG data (PTB-XL-ECG) from approximately 19,000 patients, made publicly available in 2019. Although the data were acquired previously (1989–1996), all the data were annotated in detail and the correspondence between PTB-XL-ECG and the labels was considered to have little degradation over time. There were 71 annotations consisting of 44 diagnosis-types, such as normal ECG, long QT interval, and acute myocardial infarction; 15 form-types, such as abnormal QRS and low amplitude T-waves; and 12 rhythm-types, among them sinus tachycardia and sinus bradycardia. Collectively, we referred to these 71 labels as ALL-STATEMENTS. Because monitoring the ECG involved lead II waveforms, we created models (named “PRE-MODEL”) to predict ALL-STATEMENTS (including diagnosis-types) using lead II waveforms extracted from PTB-XL-ECG. The output was a vector of length 71, indicating the labels each monitored ECG wave (MD-ECG) was likely to belong to. Then, we created a model of MD-ECG-ALL-STATEMENTS by combining each vector with the ECG data. The prediction ability of the premodel to identify ALL-STATEMENTS was high (area under the receiver operating characteristic (ROC) curve (AUROC) > 0.90) (19). We also compared the predicted probability for each label in ALL-STATEMENT between the groups with and without outcome using Student’s *t*-test.

### 2.7. Model development and internal validation

We randomly divided the patients into 3 different sets: training set (48% for model development), validation set (12% for hyperparameter tuning), and test set (40% for internal validation).

### 2.8. Model building

The model was created using Python 3.8.10, tensorflow 2.3.0, and fastai 1.0.61. The following models were trained: fully convolutional network (20), inception (21), ResNet (22), ResNeXt (23), long short-term memory (LSTM) (24), and LSTM-bidirect (25). The hyperparameters followed the open source library (19), which is a highly accurate model using PTB-XL. In addition, the frequency of the ECG was dropped from 500/s to 100/s to match PTB-XL. For the waveform of a single patient, we cut out waveforms and created multiple data entries to prevent overfitting. The outcomes of all the entries from the same patient’s monitoring ECG waveforms were uniformly set to the same outcome. Prediction accuracy generally improves with longer entries because it increases the amount of information in the time series. Therefore, we created and examined 1, 2, and 5 units of entries using a PTB-XL-ECG unit of 10 s as 1 unit. We denoted the number of units used in the entries by wave number (WVNUM). This implies that if WVNUM is equal to 5, consecutive waveforms of 50 s (5 × 10) are used as 1 entry. Because the number of entries created from the ECG monitoring varied between the patients, it was necessary to determine the maximum number of entries created per patient. We defined MAX-ENTRY as the maximum number of entries, and HALF-ENTRY as half the maximum number of entries for each WVNUM. For model development, we created 3 different experiments (a combination of data and entry): (1) Experiment 0, MD-ECG with HALF-ENTRY; (2) Experiment 1, MD-ECG-ALL-STATEMENTS with HALF-ENTRY; and (3) Experiment 2, MD-ECG-ALL-STATEMENTS with MAX-ENTR. For each experiment, we trained and predicted the all-cause mortality and CPC scores, and evaluated the performance of each WVNUM. We evaluated the average results for each patient in the test set. We used the AUROC curve for the evaluation. The code which was used in this paper is not available as an open source.

## 3. Results

### 3.1. Patient characteristics

**Figure 1:**
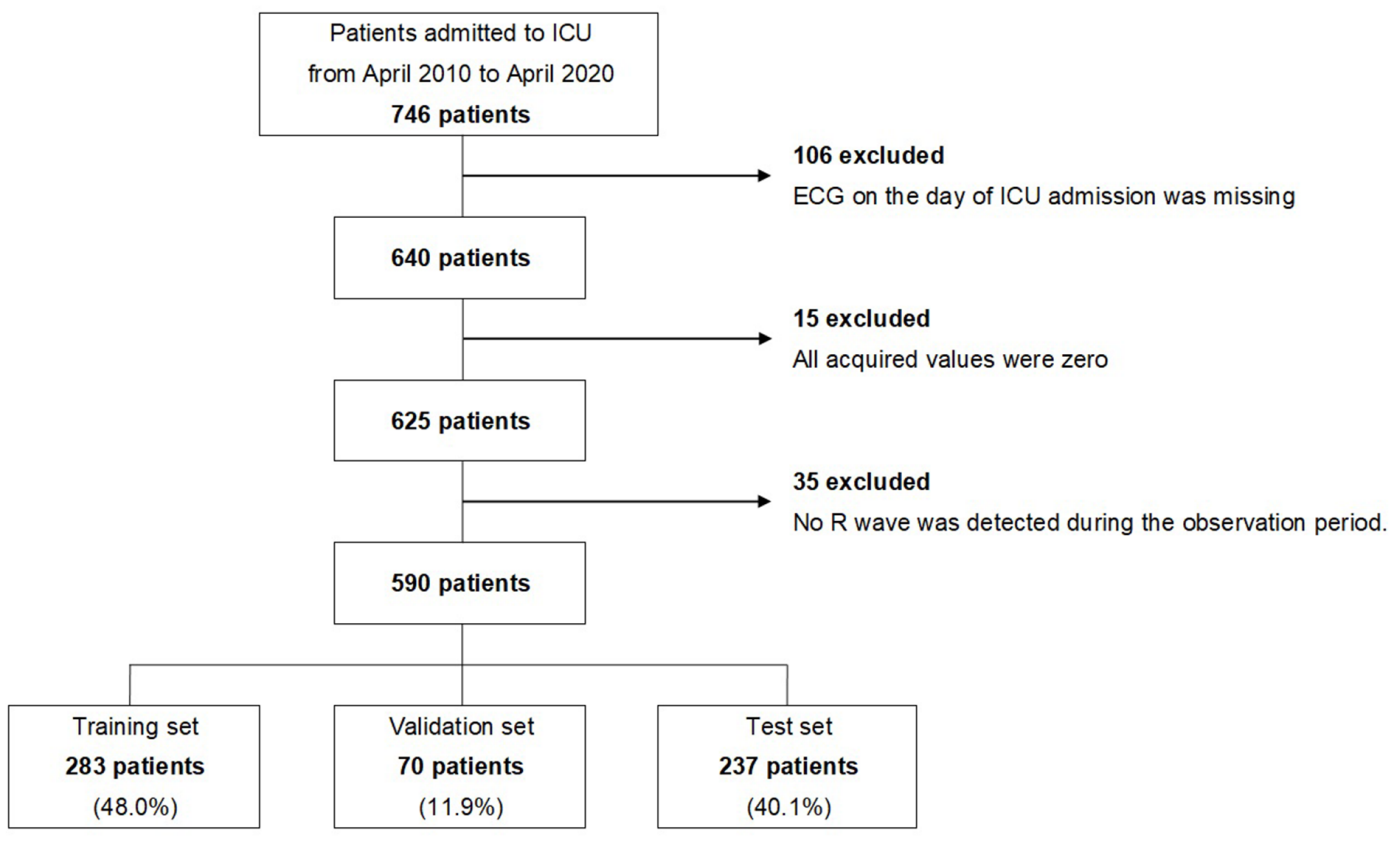
Extraction flow of the study. ICU, intensive care unit; ECG, electrocardiogram.

Between April 2010 and April 2020, 746 patients were admitted to the ICU of the Hokkaido University Hospital for OHCA. Of these, we excluded 106 patients whose monitored ECGs were not obtained on the day of admission, 15 patients whose monitored ECG values were all zero, and 35 patients whose R waves were not observed in the early stage of admission to the ICU. A total of 590 patients were finally analyzed. The extraction flow of the target patients is shown in Figure 1, and the characteristics of the patients are shown in Table 1.

**Table 1:** Baseline characteristics of the study population.

	All patients (*n* = 590)	Train set (*n* = 283)	Validation set (*n* = 70)	Test set (*n* = 237)
Age, years	71 [58–82]	68 [58–81]	72 [63–82]	73 [58–82]
Sex, male	349 (59.2)	167 (59.0)	41 (58.6)	141 (59.5)
Cause of CPA				
Internal causes				
Cardiac	306 (51.9)	147 (51.9)	40 (57.1)	119 (50.2)
Respiratory	54 (9.2)	26 (9.2)	3 (4.3)	25 (10.5)
Neurological	45 (7.6)	20 (7.1)	7 (10.0)	18 (7.6)
Other internal causes	28 (4.7)	13 (4.6)	5 (7.1)	10 (4.2)
External causes				
Asphyxia	106 (18.0)	49 (17.3)	12 (17.1)	45 (19.0)
Hanging	27 (4.6)	18 (6.4)	1 (1.4)	8 (3.4)
Drowning	5 (0.8)	2 (0.7)	1 (1.4)	2 (0.8)
Trauma	5 (0.8)	2 (0.7)	0 (0)	3 (1.3)
Other external causes	14 (2.4)	6 (2.1)	1 (1.4)	7 (3.0)
Witnessed cardiac arrest	397 (67.3)	181 (64.0)	49 (70.0)	167 (70.5)
Bystander CPR	293 (49.7)	138 (48.8)	35 (50.0)	120 (50.6)
Initial electrical rhythm				
Asystole	227 (38.5)	111 (39.2)	26 (37.1)	90 (38.0)
PEA	186 (31.5)	86 (30.4)	22 (31.4)	78 (32.9)
VF/Pulseless VT	177 (30.0)	86 (30.4)	22 (31.4)	69 (29.1)
Interval from call to EMS arrival, minutes	7 [6–8]	7 [6–8]	7 [6–8]	7 [5–8]
Interval from call to hospital arrival, minutes	35 [29–41]	35 [29–41]	35 [29–40]	35 [29–41]
Prehospital ROSC	328 (55.6)	154 (54.4)	42 (60.0)	132 (55.7)
Extracorporeal-CPR	103 (17.5)	51 (18.0)	10 (14.3)	42 (17.7)
Therapeutic hypothermia	139 (23.6)	63 (22.3)	17 (24.3)	59 (22.4)
Outcome				
Survival at discharge	326 (55.3)	159 (59.0)	38 (54.3)	129 (54.4)
Good neurological outcome (CPC 1 or 2) at discharge	97 (16.4)	44 (15.5)	14 (20.0)	39 (16.5)

All data are presented as median [interquartile range] or N (%). CPA, cardiopulmonary arrest; CPR, cardiopulmonary resuscitation; PEA, pulseless electrical activity; VF, ventricular fibrillation; VT, ventricular tachycardia; EMS, emergency medical service; ROSC, return of spontaneous circulation; CPC, cerebral performance category.

### 3.2. Predictive performance for in-hospital mortality

**Figure 2:**
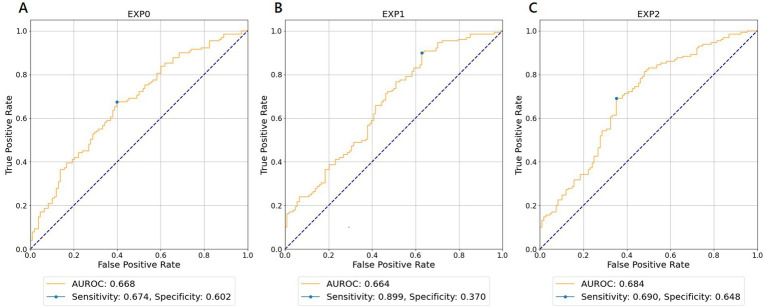
Receiver operating characteristic curves for wave number of 5 predicting in-hospital all-cause mortality. **(A)** EXP0 (MD-ECG data and HALF-ENTRY). **(B)** EXP1 (MD-ECG-ALLST data and HALF-ENTRY). **(C)** EXP2 (MD-ECG-ALLST data and MAX-ENTRY). EXP, experiment; AUROC, area under the receiver operating characteristic curve; MD-ECG, monitored ECG; ALLST, ALL-STATEMENTS.

For each experiment and WVNUM, Table 2 shows the AUROCs for in-hospital all-cause mortality. In general, the predictive ability improved with increasing WVNUM. The combination of Experiment 2 (monitoring-ECG-ALL-STATEMENTS with MAX-ENTRY) and WVNUM of 5 had the highest AUROC of 0.684 (95% confidence interval [CI], 0.680–0.689). The ROC curves for a WVNUM of 5 are shown in Figure 2. Supplementary Table 2 shows the 10 selected labels for which the predicted probability that each label in ALL-STATEMENT was significantly different between the groups with and without outcome. For example, the patients who died were less likely to be predicted to have incomplete left bundle branch block and ischemic change in the anterolateral leads. The confusion matrix is shown in Supplementary Table 4.

**Table 2:** The area under the curves (AUCs) for in-hospital all-cause mortality.

WVNUM				
		1	2	5
EXP	0	0.615 (0.612–0.619)	0.627 (0.624–0.632)	0.668 (0.662–0.673)
	1	0.627 (0.624–0.629)	0.624 (0.621–0.628)	0.664 (0.657–0.670)
	2	0.648 (0.645–0.651)	0.641 (0.638–0.644)	0.684 (0.680–0.689)

The AUCs and 95% confidence intervals are shown. WVNUM, wave number; EXP, experiment.

### 3.3. Predictive performance for neurologically favorable outcomes

**Figure 3:**
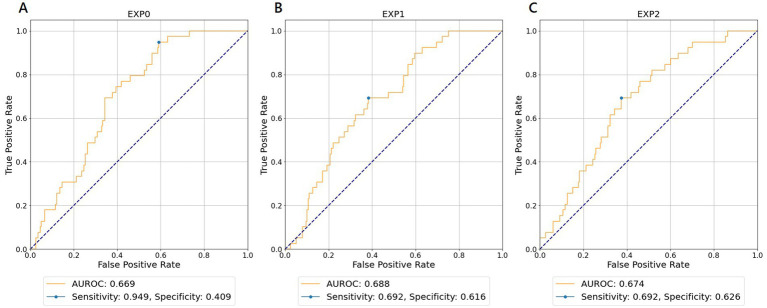
Receiver operating characteristic curves for wave number of 5 predicting favorable neurological outcomes. **(A)** EXP0 (MD-ECG data and HALF-ENTRY). **(B)** EXP1 (MD-ECG-ALLST data and HALF-ENTRY). **(C)** EXP2 (MD-ECG-ALLST data and MAX-ENTRY). EXP, experiment; AUROC, area under the receiver operating characteristic curve; MD-ECG, monitored ECG; ALLST, ALL-STATEMENTS.

Table 3 shows the results of the AUROC for neurologically favorable outcomes in each experiment and WVNUM. In general, the predictive ability improved with increasing WVNUM. The combination of Experiment 1 (monitoring-ECG-ALL-STATEMENTS with HALF-ENTRY) and WVNUM of 5 had the highest AUROC of 0.688 (95% CI, 0.682–0.694). The ROC curves for a WVNUM of 5 are shown in Figure 3. Supplementary Table 3 shows the 10 selected labels that have a high predictive probability of neurological outcome among ALL-STATEMENT labels. Patients with a worse neurological outcome were less likely to be predicted to have ECG changes suggesting lateral myocardial infarction, right ventricular hypertrophy, and electrolytic disturbance or drug. The confusion matrix is shown in Supplementary Table 4.

**Table 3:** The area under the curves (AUCs) for favorable neurological outcomes.

WVNUM				
		1	2	5
EXP	0	0.581 (0.578–0.584)	0.654 (0.650–0.659)	0.669 (0.664–0.675)
	1	0.576 (0.565–0.588)	0.626 (0.620–0.632)	0.688 (0.682–0.694)
	2	0.586 (0.571–0.600)	0.550 (0.546–0.553)	0.674 (0.666–0.681)

The AUCs and 95% confidence intervals are shown. WVNUM, wave number; EXP, experiment.

## 4. Discussion

In this study, we demonstrated the feasibility of using the continuous ECG monitoring waveform, which was not a 12-lead ECG, for 1 h soon after admission to the ICU to predict survival and neurological outcomes in patients with OHCA using ML techniques. To our knowledge, this is the first study to use this method. In our model, the highest values of AUROC were 0.684 and 0.688 for survival and neurological outcome, respectively, which indicated the possibility of using the waveform of continuous ECG monitoring as a predictor in patients with OHCA.

ECG waveforms contain various types of information, such as rhythm, rate, conduction, hypertrophy, and ischemia. Serious ECG abnormalities are directly associated with life-threatening conditions. Several studies have reported the relationship between 12-lead ECG abnormalities after return of spontaneous circulation (ROSC) and survival outcomes in patients with OHCA (26, 27). A study showed that a long QRS duration of the 12-lead ECG at ROSC and after therapeutic hypothermia was associated with a high mortality rate (26). Another study reported that an accelerated idioventricular rhythm of the 12-lead ECG at ROSC was associated with a lower survival rate at 7 days (27). However, these results only showed a relationship between ECG abnormalities due to severe cardiac dysfunction and mortality in patients with OHCA.

Predicting neurological outcomes using ECG waveforms is a challenge. In patients with traumatic brain injury (TBI), HRV, measured by the variation in the R-R intervals, was reported to be a predictor of neurological outcomes (28). In patients with OHCA, Endoh et al. (8) analyzed the HRV of the monitored ECG during hypothermic targeted temperature management within 24 h after ROSC and showed that HRV could be useful for neurological prognosis. Because HRV is affected by the patients’ autonomic function (28), it could have a neurological predictive value in patients with TBI or OHCA. Another study on 12-lead ECG in patients with OHCA reported that a lower QT and a corrected dispersion of the QT interval 24 h after ROSC was associated with survival and neurological outcomes (10). The authors believed the sympathetic innervation of the left ventricle was damaged by myocardial ischemia, which caused dispersion of the QT interval. These previous studies (8, 10) were designed to analyze ECG waveforms at or within 24 h after ROSC. Our model indicated the predictive value of continuous ECG waveforms for 1 h shortly after admission in patients with all-cause OHCA.

Previous prognostic scores in patients with OHCA (3–6) showed high predictive power, but some of them were not always applicable, especially in patients without witnesses due to the lack of information. The current guidelines for resuscitation and post-resuscitation care published by the American Heart Association (29), the European Resuscitation Council and the European Society of Intensive Care Medicine (30) recommend that a prognostic assessment should be performed with a multimodal strategy using clinical examination (pupillary reflex, pupillometry, corneal reflex myoclonus, and the Glasgow Motor Score), neurophysiology (electroencephalography and somatosensory evoked potentials), biomarkers [neuron-specific enolase (NSE)], and imaging [computed tomography (CT) and magnetic resonance imaging (MRI)]. According to the guidelines, clinicians should make these assessments at 24, 48, and 72 h after ROSC; therefore, there is little information on neurological prognosis immediately after admission. For example, NSE was a strong neurological predictor, and NSE at admission and day 3 had AUROC values of 0.68 and 0.89, respectively (31). Another neurological predictor, specifically the grey-matter to white-matter ratio (GWR) measured on brain CT, had an AUROC value of 0.91 in a previous meta-analysis (32). However, in a study investigating brain CT images within 1 h after ROSC, the GWR had an AUROC value of 0.685–0.747 (33). Therefore, assessing the neurological prognosis of patients with OHCA at admission is difficult, and the present study identified a possible new predictor.

Simple 3-electrode monitoring is widely used in various situations for continuous ECG monitoring. In this study, we used II-lead waveforms of continuous ECG monitoring in the ICU, because P waves, QRS complexes, and T waves are recognizable in II-lead waveforms. Unlike the 12-lead ECG, 3-electrode monitoring ECG does not have a standard for electrode placement, and clinicians or nurses may place electrodes at imprecise positions on the patient’s body. However, we showed that the ECG waveforms had a predictive value. ECG monitoring is an essential non-invasive component for patients in critical conditions. Therefore, clinicians can obtain monitored ECG information without additional effort or cost. Furthermore, it can be expected to perform prehospital ECG analysis in the future. It is important to note that the neurologic prognosis of patients with OHCA should be assessed 72 h after resuscitation (29, 30) and should not be determined at admission.

Using deep learning for ECG data has recently increased. Most studies have used standard short-duration 12-lead ECG data to diagnose arrhythmias (34–36), 1-year mortality rate (37), or automatic triage based on medical records (38). On the contrary, to our knowledge, few studies have used long-duration monitoring ECG data because they are highly resource-consuming (processor and memory) and require long-time durations for learning. We trained the deep learning model on 1 h of ECG monitoring data by separating the training data into small segments and integrating the results to achieve resource-optimized computation.

This study had several limitations. First, this was a single-center retrospective study, and ECG monitoring devices from the same manufacturer were used for all the patients. However, the ECG monitoring waveform does not differ substantially between the devices. Second, we did not examine the factors that might influence the ECG waveforms such as drugs or underlying diseases. Third, although we included the patients with all-cause OHCA in this study (because of the limited sample size), patients having cardiac-originating conditions tended to show different waveforms from those having noncardiac-originating conditions, because many of the former have heart rhythm or conduction abnormalities. Fourth, our model showed the probability of predicting short-term outcomes in patients with OHCA. However, long-term outcomes were not analyzed. Finally, external validation is needed to confirm the association between monitoring ECG data and outcomes in patients with OHCA. However, given the potential underlying mechanisms, our findings should facilitate the use of routinely collected ECG monitoring data to predict prognosis among patients with OHCA.

## 5. Conclusion

Our model, supported by ML, demonstrated the possibility of using monitored ECG waveforms to predict survival and neurological outcomes in patients with OHCA. A large-scale multicenter prospective validation study should be performed for a wider application of this model. The predictive performance of our model was limited, however, it could be increased by combining monitored ECG waveforms and other variables.

## Data availability statement

The raw data supporting the conclusions of this article will be made available by the authors, without undue reservation.

## Ethics statement

The studies involving human participants were reviewed and approved by Ethics Committee of Hokkaido University Hospital. Written informed consent for participation was not required for this study in accordance with the national legislation and the institutional requirements.

## Author contributions

MH and TG planned and designed the study and revised the manuscript. MH collected the data. TG and KO analyzed the data. MT and KO drafted the original manuscript. All authors contributed to the article and approved the submitted version.

## Conflict of interest

The authors declare that the research was conducted in the absence of any commercial or financial relationships that could be construed as a potential conflict of interest.

## Publisher’s note

All claims expressed in this article are solely those of the authors and do not necessarily represent those of their affiliated organizations, or those of the publisher, the editors and the reviewers. Any product that may be evaluated in this article, or claim that may be made by its manufacturer, is not guaranteed or endorsed by the publisher.

## Abbreviations

CPR: cardiopulmonary resuscitation
OHCA: out-of-hospital cardiac arrest
CA: cardiac arrest
ECG: electrocardiogram
HRV: heart rate variability
ML: machine learning
ICU: intensive care unit
CPC: cerebral performance category
LSTM: long short-term memory
MD-ECG: monitored ECG
WVNUM: wave number
ROC: receiver operating characteristic
AUROC: area under the receiver operating characteristic curve
ROSC: return of spontaneous circulation
TBI: traumatic brain injury
CT: computed tomography
