# Supplementary material for: Electrocardiogram monitoring as a predictor of neurological and survival outcomes in patients with out-of-hospital cardiac arrest: a single-center retrospective observational study
Source: Front Neurol. 2023 Jul 4;14:1210491. doi: 10.3389/fneur.2023.1210491 (PMC10352613; doi:10.3389/fneur.2023.1210491)
Supplement: Supplementary file 1 [file Table_1.DOCX]

Supplementary Material

Electrocardiogram monitoring as a predictor of neurological and survival outcomes in patients with out-of-hospital cardiac arrest: A single-centre retrospective observational study

Masaki Takahashi, Kentaro Ogura, Tadahiro Goto, Mineji Hayakawa*

*** Correspondence:** Mineji Hayakawa: mineji@dream.com

|  | Wave value (μV) | Count | Proportion (%) |  |
| --- | --- | --- | --- | --- |
|  | v < 0 | 5,272 | 0.00 |  |
|  | v = 0 | 135,774,267 | 8.32 |  |
|  | 0 < v ≤ 1000 | 13,572 | 0.00 |  |
|  | 1000 < v ≤ 2000 | 11,749 | 0.00 |  |
|  | 2000 < v ≤ 3000 | 15,196 | 0.00 |  |
|  | 3000 < v ≤ 4000 | 22,150 | 0.00 |  |
|  | 4000 < v ≤ 5000 | 32,755 | 0.00 |  |
|  | 5000 < v ≤ 6000 | 59,677 | 0.00 |  |
|  | 6000 < v ≤ 7000 | 159,127 | 0.01 |  |
|  | 7000 < v ≤ 8000 | 8,457,497 | 0.52 |  |
|  | 8000 < v ≤ 9000 | 1,487,268,628 | 91.11 |  |
|  | 9000 < v ≤ 10000 | 349,677 | 0.02 |  |
|  | v > 10000 | 185,478 | 0.01 |  |

**Supplementary Table 1.** Wave value range and data count.
