# Supplementary material for: Electrocardiogram monitoring as a predictor of neurological and survival outcomes in patients with out-of-hospital cardiac arrest: a single-center retrospective observational study
Source: Front Neurol. 2023 Jul 4;14:1210491. doi: 10.3389/fneur.2023.1210491 (PMC10352613; doi:10.3389/fneur.2023.1210491)
Supplement: Supplementary file 2 [file Table_2.DOCX]

Supplementary Material

Electrocardiogram monitoring as a predictor of neurological and survival outcomes in patients with out-of-hospital cardiac arrest: A single-centre retrospective observational study

Masaki Takahashi, Kentaro Ogura, Tadahiro Goto, Mineji Hayakawa*

*** Correspondence:** Mineji Hayakawa: mineji@dream.com

|  |  | Predicted probability (‰) | | |  |  |
| --- | --- | --- | --- | --- | --- | --- |
|  |  | Overall | Survived | Died |  |  |
|  | Description (label) | n=590 | n=326 | n=264 | p-value |  |
|  | Incomplete right bundle branch block | 0.8 (0.2) | 0.8 (0.3) | 0.8 (0.2) | 0.001 |  |
|  | Incomplete left bundle branch block | 8.0 (0.4) | 8.0 (0.4) | 7.9 (0.5) | 0.001 |  |
|  | Prolonged PR interval | 0.3 (0.0) | 0.3 (0.1) | 0.3 (0.0) | 0.001 |  |
|  | Premature complex(es) | 16.1 (2.2) | 16.4 (2.5) | 15.8 (1.7) | 0.001 |  |
|  | Digitalis-effect | 48.6 (9.2) | 49.6 (9.9) | 47.3 (8.1) | 0.002 |  |
|  | Non-specific intraventricular conduction disturbance (block) | 117.3 (12.2) | 118.7 (12.6) | 115.6 (11.5) | 0.002 |  |
|  | Ischaemic in anterolateral leads | 38.2 (4.6) | 38.7 (4.3) | 37.5 (4.8) | 0.002 |  |
|  | Pattern of lateral myocardial infarction | 1.8 (0.2) | 1.9 (0.2) | 1.8 (0.2) | 0.003 |  |
|  | Low QRS voltages in the frontal and horizontal leads | 42.2 (8.6) | 43.1 (9.8) | 41.1 (6.9) | 0.003 |  |
|  | Low amplitude T-waves | 24.8 (6.6) | 25.5 (7.5) | 24.0 (5.1) | 0.004 |  |

**Supplementary Table 2.** The selected labels for which the predicted probability that each label in ALL-STATEMENT was significantly.

Values were predicted probability (‰) with standard deviation for each label in ALL-STATEMENT (the overall number of labels is 71).

P-values were calculated using t-test.
