# Supplementary material for: Electrocardiogram monitoring as a predictor of neurological and survival outcomes in patients with out-of-hospital cardiac arrest: a single-center retrospective observational study
Source: Front Neurol. 2023 Jul 4;14:1210491. doi: 10.3389/fneur.2023.1210491 (PMC10352613; doi:10.3389/fneur.2023.1210491)
Supplement: Supplementary file 3 [file Table_3.DOCX]

Supplementary Material

Electrocardiogram monitoring as a predictor of neurological and survival outcomes in patients with out-of-hospital cardiac arrest: A single-centre retrospective observational study

Masaki Takahashi, Kentaro Ogura, Tadahiro Goto, Mineji Hayakawa*

*** Correspondence:** Mineji Hayakawa: mineji@dream.com

|  |  | Predicted probability (‰) | | |  |  |
| --- | --- | --- | --- | --- | --- | --- |
|  |  | Overall | CPC 1-2 | CPC 3-5 |  |  |
|  | Description (label) | n=590 | n=97 | n=493 | p-value |  |
|  | Pattern of lateral myocardial infarction | 1.8 (0.2) | 1.9 (0.1) | 1.8 (0.2) | <0.001 |  |
|  | Pattern of Right ventricular hypertrophy | 43.6 (2.0) | 44.0 (1.0) | 43.5 (2.2) | 0.001 |  |
|  | Electrolytic disturbance or drug (former EDIS) | 7.7 (1.0) | 7.9 (0.6) | 7.6 (1.0) | 0.001 |  |
|  | Pattern of inferoposterolateral myocardial infarction | 53.8 (4.4) | 54.7 (3.2) | 53.7 (4.6) | 0.007 |  |
|  | Wolf-Parkinson-White syndrome | 7.4 (1.2) | 7.6 (1.0) | 7.4 (1.3) | 0.019 |  |
|  | Incomplete left bundle branch block | 8.0 (0.4) | 8.1 (0.2) | 8.0 (0.5) | 0.023 |  |
|  | Normal functioning artificial pacemaker | 0.6 (0.1) | 0.6 (0.1) | 0.6 (0.1) | 0.032 |  |
|  | Sinus bradycardia | 134.6 (7.9) | 135.9 (6.3) | 134.4 (8.1) | 0.036 |  |
|  | Left posterior fascicular block | 55.7 (5.6) | 56.6 (4.3) | 55.5 (5.9) | 0.037 |  |
|  | Ischaemic in anterolateral leads | 38.2 (4.6) | 39.0 (4.0) | 38.1 (4.7) | 0.042 |  |

**Supplementary Table 3.** The selected labels for which the predicted probability that each label in ALL-STATEMENT was significantly.

Values were predicted probability (‰) with standard deviation for each label in ALL-STATEMENT (the overall number of labels is 71).

P-values were calculated using t-test.

Abbreviations: CPC, cerebral performance categories
