# Supplementary material for: Electrocardiogram monitoring as a predictor of neurological and survival outcomes in patients with out-of-hospital cardiac arrest: a single-center retrospective observational study
Source: Front Neurol. 2023 Jul 4;14:1210491. doi: 10.3389/fneur.2023.1210491 (PMC10352613; doi:10.3389/fneur.2023.1210491)
Supplement: Supplementary file 4 [file Table_4.DOCX]

Supplementary Material

Electrocardiogram monitoring as a predictor of neurological and survival outcomes in patients with out-of-hospital cardiac arrest: A single-centre retrospective observational study

Masaki Takahashi, Kentaro Ogura, Tadahiro Goto, Mineji Hayakawa*

*** Correspondence:** Mineji Hayakawa: mineji@dream.com

|  |  |  | Predicted | | | |  |
| --- | --- | --- | --- | --- | --- | --- | --- |
|  |  |  | All death | | CPC 1-3 | |  |
|  |  |  | Negative | Positive | Negative | Positive |  |
|  | Actual | Negative | 22 | 86 | 194 | 4 |  |
|  |  | Positive | 7 | 122 | 37 | 2 |  |

**Supplementary Table 4.** Confusion matrix of the developed models.

All data are presented as number.

Abbreviations: CPC, cerebral performance categories
